# Uncovering the alarming rise of diabetic ketoacidosis during COVID-19 pandemic: a pioneer African study and review of literature

**DOI:** 10.3389/fendo.2023.1234256

**Published:** 2023-07-26

**Authors:** Asma Gorchane, Taieb Ach, Jihene Sahli, Asma Ben Abdelkrim, Manel Mallouli, Foued Bellazreg, Wissem Hachfi, Molka Chadli Chaieb, Koussay Ach

**Affiliations:** ^1^ Department of Endocrinology, University Hospital of Farhat Hached, Sousse, Tunisia; ^2^ University of Sousse, Faculty of Medicine of Sousse, Sousse, Tunisia; ^3^ Laboratory of Exercice Physiology and Pathophysiology, Tunis, Tunisia; ^4^ Department of Community Medicine, University Hospital of Farhat Hached, Sousse, Tunisia; ^5^ Department of Infectious Diseases, University Hospital of Farhat Hached, Sousse, Tunisia

**Keywords:** diabetic ketoacidosis, COVID-19, type 1 diabetes, type 2 diabetes, epidemiology

## Abstract

**Introduction:**

Reports around the world indicate that COVID-19 pandemic may be contributing to an increase in the incidence of new onset diabetic ketoacidosis (DKA). This has yet to be studied in Africa. We aimed to compare the incidence trend of new onset DKA before and during the COVID-19 pandemic, with a focus on the type of diabetes mellitus (DM).

Materials and methods

This was a cross sectional analytical study, over a 4-year period, between March 2018 until February 2022 conducted in the referral center: diabetology department of university hospital Farhat Hached Sousse, Tunisia. The study population included patients hospitalized for new onset DKA divided in two groups: G1: before COVID-19 pandemic and G2: during COVID-19 pandemic. Patients younger than 14, new onset DM not presenting with DKA, other types of diabetes (monogenic, secondary or pancreatic diabetes) were not included. A statistical analysis of the monthly incidence trend was conducted using the Jointpoint software providing the average monthly percentage of change (AMPC).

**Results:**

a total of 340 patients were included:137 registered before the pandemic and 203 during the pandemic, representing a 48.17% increase. The mean monthly incidence of new onset DKA during COVID-19 pandemic was statistically higher than that before COVID-19 pandemic (8.42 ± 4.87 vs 5.75 ± 4.29 DKA per month) (p=0.049). The temporal trend of DKA during the 4-year study showed a significant upward trend with a change in AMPC of +0.2% (p=0.037). The incidence of type 1 diabetes (T1D) and type 2 diabetes (T2D) increased by 50% and 44% respectively during COVID-19 pandemic. Anti-glutamic acid decarboxylase (anti-GAD) antibodies’ titers significantly increased in G2 compared with G1 (median of 330[Q1–Q3]=[58.5–1795]vs 92.5[Q1–Q3]=[22.5–1074] respectively)(p=0.021).

**Discussion:**

The incidence trend of DKA showed an increase during the COVID-19 pandemic along with an increase of T1D and T2D implying that the pandemic may have been the underlying factor of this upward trend.

## Introduction

The COVID-19 pandemic has had a profound impact on global health systems, overwhelming hospitals and healthcare workers with an unprecedented influx of patients (1). Africa was not spared from the rapid propagation of the disease adding to the burden of a precarious health system already plagued by endemic diseases (2). Reports from various regions of the world have suggested a possible association between COVID-19 and the development of new-onset diabetic ketoacidosis (DKA) (3).

DKA is a potentially life-threatening complication of diabetes mellitus (DM) affecting both type 1 (T1D) and type 2 diabetes (T2D) (4). Its incidence has been increasing over the last few decades, which has been attributed to a combination of factors, mainly the overall rising prevalence of DM (5). Furthermore, recent studies have suggested an additional increase in DKA during the COVID-19 pandemic, raising speculations about a potential involvement of COVID-19, whether it is directly or indirectly, in this upward trend (6–8).

On the one hand, the pandemic may have caused delays in diagnosis for fear of contracting the virus resulting in higher DKA cases (9). On the other hand, SARS-CoV-2 may have caused direct damage to pancreatic cells (10), triggered auto-immunity (11) or promoted insulin resistance (12). This underscores the relevance of investigating COVID-19’s role in new onset DKA.

Our study sought to compare epidemiological aspects of new onset DKA including incidence of DKA specifically focusing on T1D and T2D, before and during COVID-19 pandemic within the context of an African country, aiming to provide valuable insights into the complex interplay between COVID-19 and new onset DM.

## Materials and methods

### Study design and setting

We conducted a cross-sectional descriptive and analytical study carried out in the Diabetology & Endocrinology department of Farhat Hached University Hospital of Sousse.

### Inclusion criteria

It has included all the patients who had been hospitalized for new onset DKA over a period of 4 years between the year 2018 and 2022, that is before and during COVID-19 pandemic.

Since the second of March 2020 was the mark of patient zero in Tunisia declaring the start of the pandemic in Tunisia (13), the population was automatically divided in two groups:

- Group1 (G1): patients hospitalized before COVID-19 pandemic since the first of March of 2018 until first of March 2020.
- Group2 (G2): patients hospitalized during COVID-19 pandemic since second of March 2020 until 28th February 2022.

### Non-inclusion criteria

Patients younger than 16 years old, patients with known DM, patients with new onset DM not presenting with DKA were not included in this study.

### Exclusion criteria

All other types of diabetes (high presumption of monogenic diabetes, gestational, secondary or pancreatic diabetes) were excluded from this study.

We note that our study did not include genetic testing to specifically exclude monogenic diabetes. Instead, we excluded patients with a strong likelihood of monogenic diabetes based on clinical assessments and criteria (14).

### Data collection

Data collection was conducted retrospectively by filling in a standardized information sheet collected by consulting medical records coded as “new onset DKA” for patients who were hospitalized two years before and during the COVID-19 pandemic.

### Variables

Epidemiological data was compared between the two groups including incidence, the date of admission, month and season of discovery, duration of polyuria and polydipsia.

Islet antibodies and c-peptide were measured using the enzyme-linked immunosorbent assay (ELISA) in all patients at admission and their respective titers were compared between the two groups. In light of clinical and biological data such as islet antibodies and C-peptide, the new onset DM was eventually classified as T1D or T2D. The types of DM were compared between the two groups. Precipitating factors were compared between the two groups. It can include (15):

1-cardiovascular factors: myocardial infarction, stroke.
2-infections (urinary, pulmonary, COVID-19, otolaryngological, cutaneous, profound).
3-drugs that affect carbohydrate metabolism, such as corticosteroids.
4-psychological factors.
5-Excessive food intake.

The incidence of new onset DKA during COVID-19 pandemic was analyzed according to the pandemic waves which were specified according to National Observatory of New and Emerging Diseases (ONMNE) (16) as follows

1-First wave: from 17/08/2020 to 13/12/2020.
2-Second wave: from 14/12/2020 to 21/03/2021.
3-Third wave: 22/03/2021 to 16/05/2021.
4-Fourth wave: 17/05/2021 to 14/11/2021.
5-The fifth wave: 15/11/2021 to 28/02/2022.

The average monthly admission of DKA during COVID-19 according to the different waves were calculated and compared.

Time for DKA resolution expressed in hours, cumulative insulin dose as well as mean weight-based insulin at discharge were compared before and during COVID-19 pandemic.

### Statistical analysis

The analysis of the incidence trend variations of DKA was performed using the JOINPOINT Version 116 4.5.0.1 software. Monthly data was used, and the software provided the monthly percentage of change (MPC and AMPC: Monthly Percent Change and Average Monthly Percent Change) with a 95% confidence interval. Data were analyzed using SPSS 26.0 software. Average monthly admission of DKA during the different pandemic waves were compared using ANOVA test. Quantitative variables were presented by means and standard deviation (SD) or median and quartiles [Q1–Q3] according to the normality of the distribution which was tested using Kolmogorov-Smirnov test. Our study respected all standards in ethics in research. The anonymity and data confidentiality of the patients’ data were respected. We obtained approval from the Ethical Committee of University of Medicine of Sousse for our study, with the assigned number 4935/2023.

## Results

A total of 340 patients were included. G1 counted 137 patients while G2 counted 203 patients.

The number of DKA cases witnessed an increase of 48.17% over a similar time interval.

The mean monthly incidence of DKA before COVID-19 pandemic (G1) was statistically different from that observed during COVID-19 pandemic (G2) with a mean of 5.75 ± 4.29 DKA per month in G1 vs 8.42 ± 4.87 DKA per month (p=0.049).

**Figure 1:**
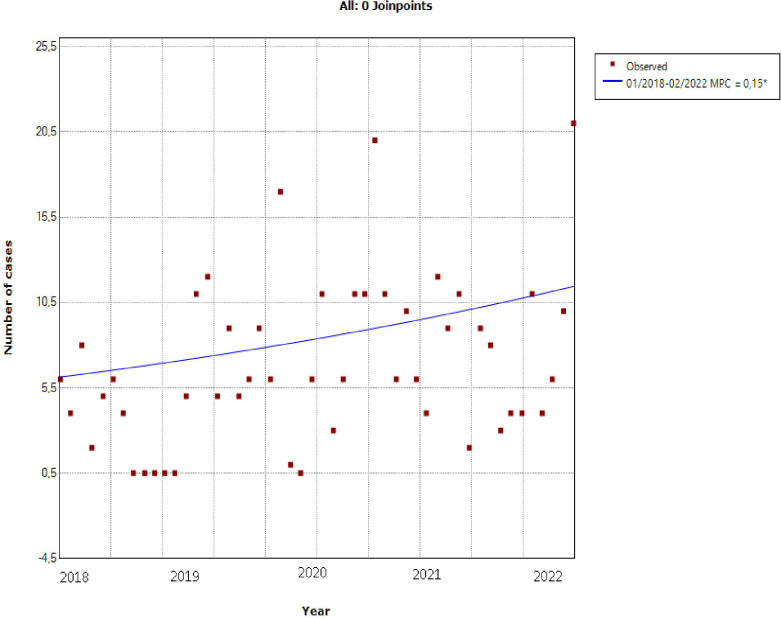
Temporal trend of hospital DKA between March 2018 and February 2022: it shows a significant upward trend with a change in the average monthly percent change (AMPC) of +0.2%, with p=0.037.

The study of the temporal trend of hospital cases of DKA between March 2018 and February 2022 showed a significant upward trend with a change in the average monthly percent change (AMPC) of +0.2%, with p=0.037 (**Figure 1**, **Table 1**).

**Table 1:** Monthly percent changes of new DKA cases.

Monthly Percent Change (MPC)							
Segment	Lower Endpoint	Upper Endpoint	MPC	Lower CI	Upper CI	Test Statistic (t)	Prob > ǀtǀ
1	01/2018	2022	0.2*	0.0	0.3	2.1	0.037
*Indicates that the monthly Percent Change (MPC) is significantly different from zero at the alpha =0.05 level.							
***Average Monthly Percent (AMPC)***							
Range	Lower Endpoint	Upper Endpoint	AMPC	Lower CI	Upper CI	Test Statistic	P-Value~
2018-2022	01/2018	2022	0.2*	0.0	0.3	2.1	<0.1
*Indicates that the AMPC is significantly different from zero at the alpha=005 level. **~ If the AMPC is within one segment, the t-distribution is used. Otherwise, the normal (z) distribution is used.**							

**Figure 2:**
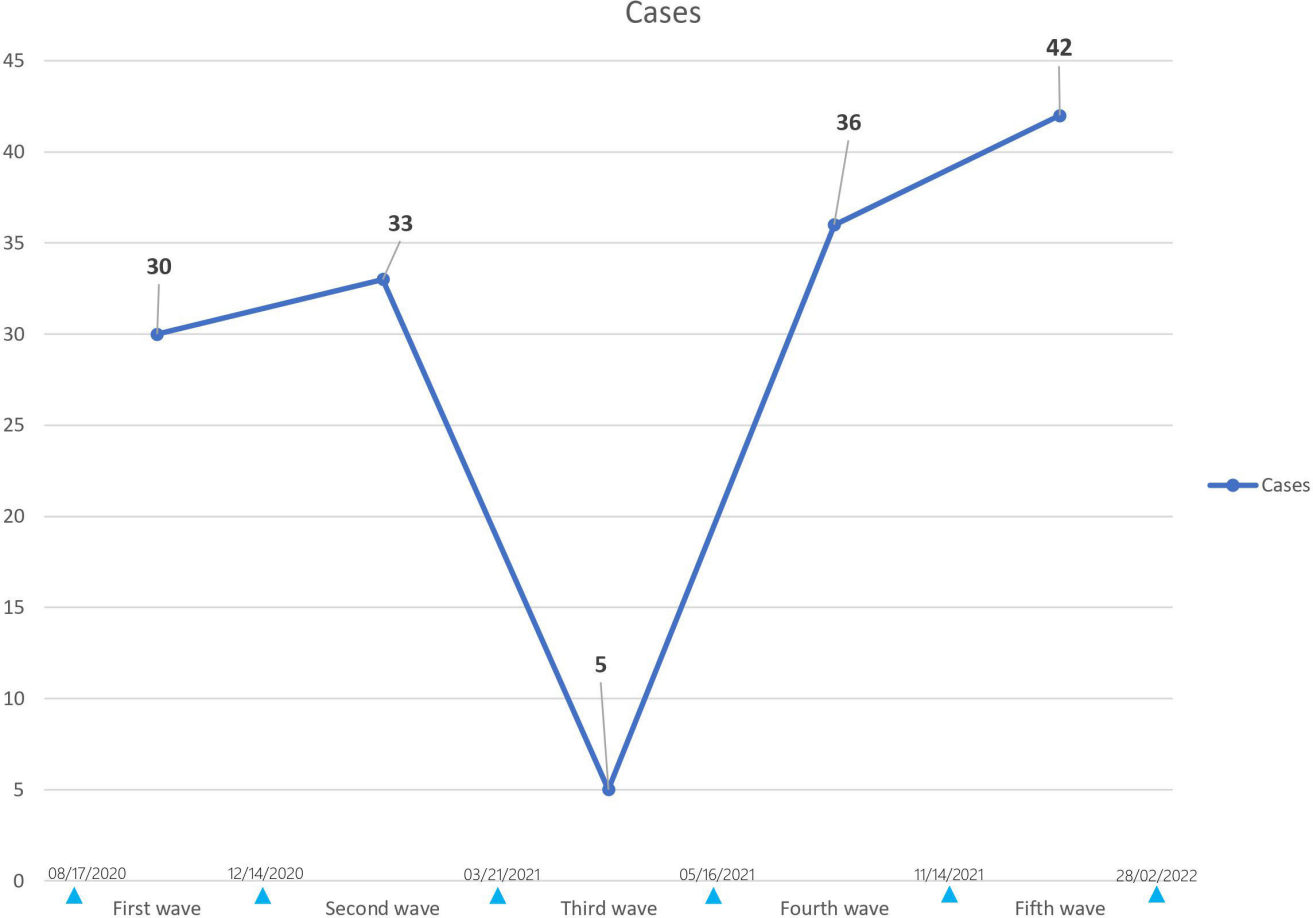
Incidence of DKA during COVID-19 according to pandemic waves: it shows a decrease during the third wave with a re-increase during the fourth and fifth waves.

The incidence of DKA according to pandemic waves during COVID-19 pandemic shows a decrease during the third wave with a re-increase during the fourth and fifth waves (**Figure 2**).

The average monthly admissions during each pandemic wave were as follows: 9.9 DKA/month for the first wave, 17 DKA/month for the second wave, 2.75 DKA/month for the third wave, 6 DKA/month for the fourth wave, and 12.5 DKA/month for the fifth wave. DKA admissions decreased during the third wave and increased during the fifth wave but the difference was not statistically significant with p=0.09.

**Figure 3:**
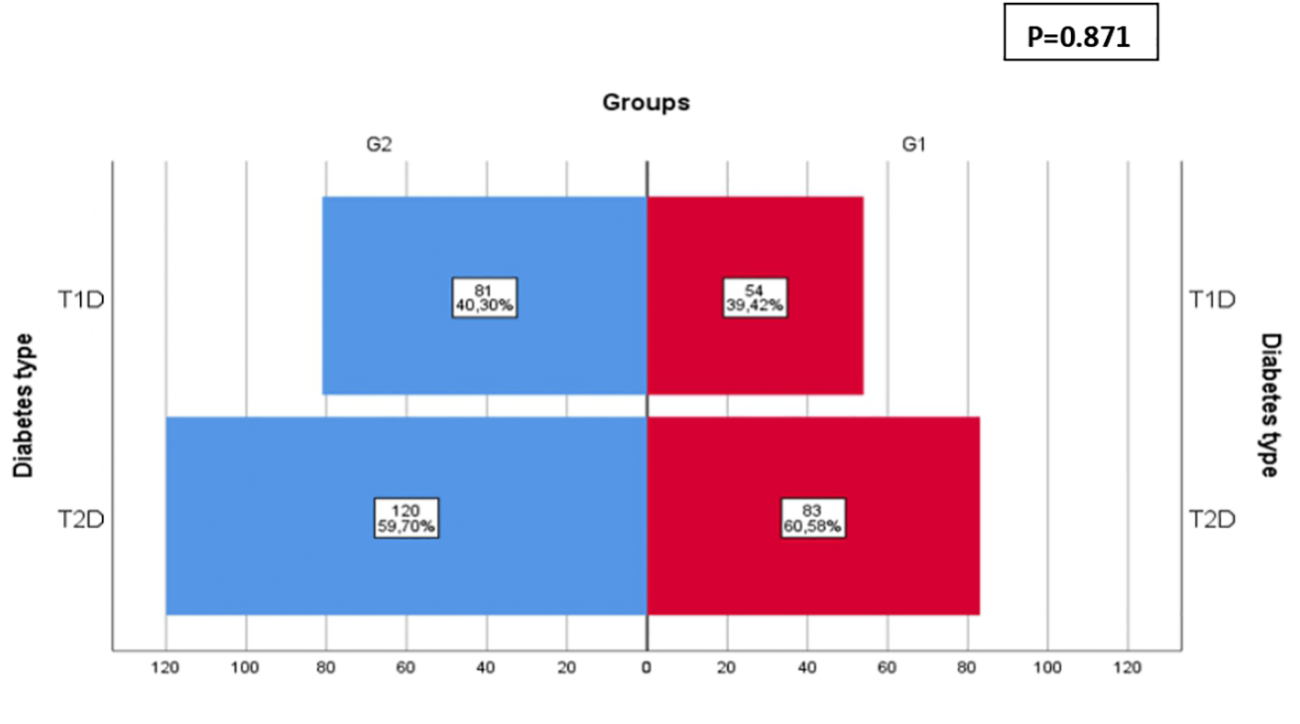
Distribution of diabetes type before (GI) and during COVID-19 (G2). The incidence of TID increased by 50% during COVID-19 pandemic as 54 (38.42%) out of 137 patients were TID in G1 vs 81 (40.30%) out of 201 patients in G2 while the incidence of T2D increased by 44% as 83 (60.58%) out of 137 patients were T2D in GI vs 120 (59.70%) out of 201 patients in G2. However, the distribution of TID and T2D frequencies was comparable between the two groups (p=0.871).

The incidence of T1D increased by 50% during COVID-19 pandemic as 54 (38.42%) out of 137 patients were T1D in G1 vs 81 (40.30%) out of 201 patients in G2 while the incidence of T2D increased by 44% as 83 (60.58%) out of 137 patients were T2D in G1 vs 120 (59.70%) out of 201 patients in G2. However, the distribution of T1D and T2D frequencies was comparable between the two groups (p=0.871) (**Figure 3**).

**Figure 4:**
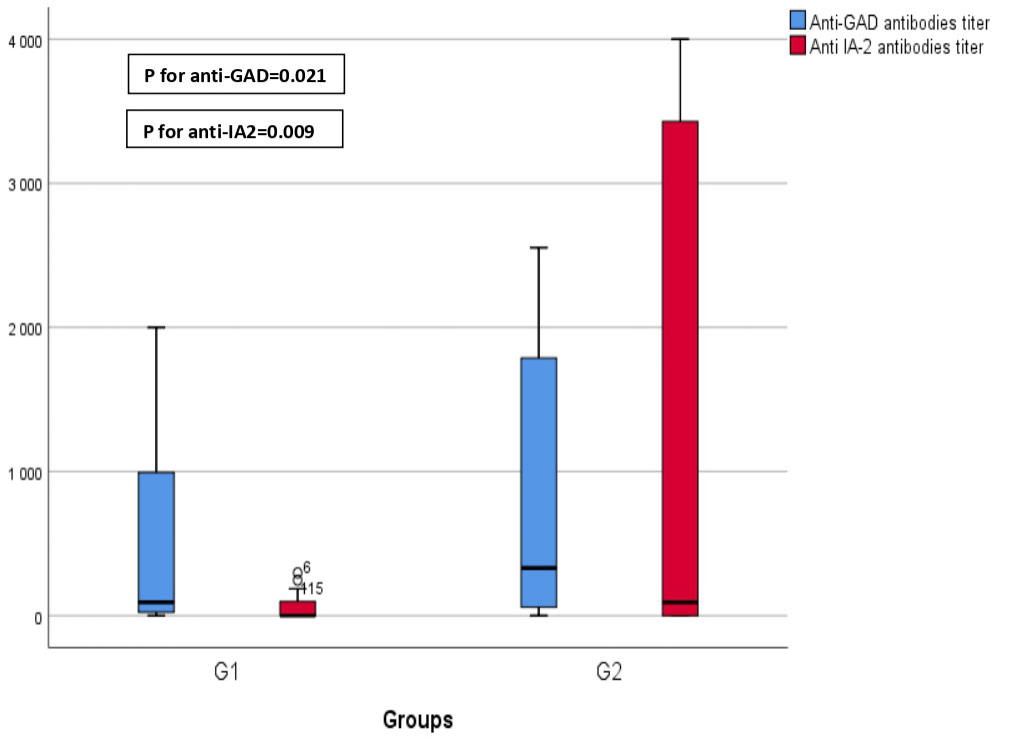
Comparison of antibodies' titers before (GI) and during COVID-19 (G2). This graphical representation provides a comparison of anti-glutamic acid decarboxylase (anti-GAD) and anti-islet cell antigen (anti-IA2) titers before and during the COVID-19 pandemic. The data reveals a statistically significant increase in anti-GAD titers during COVID-19 (GI) compared to the prepandemic period (p=0.021). Furthermore, it demonstrates a significant elevation in anti-IA2 titers during COVID-19 (G2) in contrast to the prepandemic period (p=0.009).

Anti-glutamic acid decarboxylase (Anti-GAD) antibodies titers significantly increased during the pandemic period compared with the pre-pandemic period with a median value of 92.5 [Q1–Q3]=[22.5–1074] in G1 vs 330 [Q1–Q3]=[58.5–1795] in G2 (**p=0.021**) (**Figure 4**).

Anti-islet cell antigen (Anti-IA2) antibodies titers significantly increased as well during the pandemic period compared with the pre-pandemic period with a median value of 0 [Q1–Q3]=[0–104.75] in G1 vs 93 [Q1–Q3]=[0–3571] in G2 (p=**0.009**) (**Figure 4**).

The main precipitating factors of DKA before and during COVID-19 pandemic were comparable between the two groups: infections (29.10% in G1 vs 28.64% in G2), excessive food intake (2.24% in G1 vs 6.53% in G2), psychological stress (22.39% in G1 vs 31.16% in G2), no precipitating factor was found in 40.3% vs 31.66% in G2.

**Figure 5:**
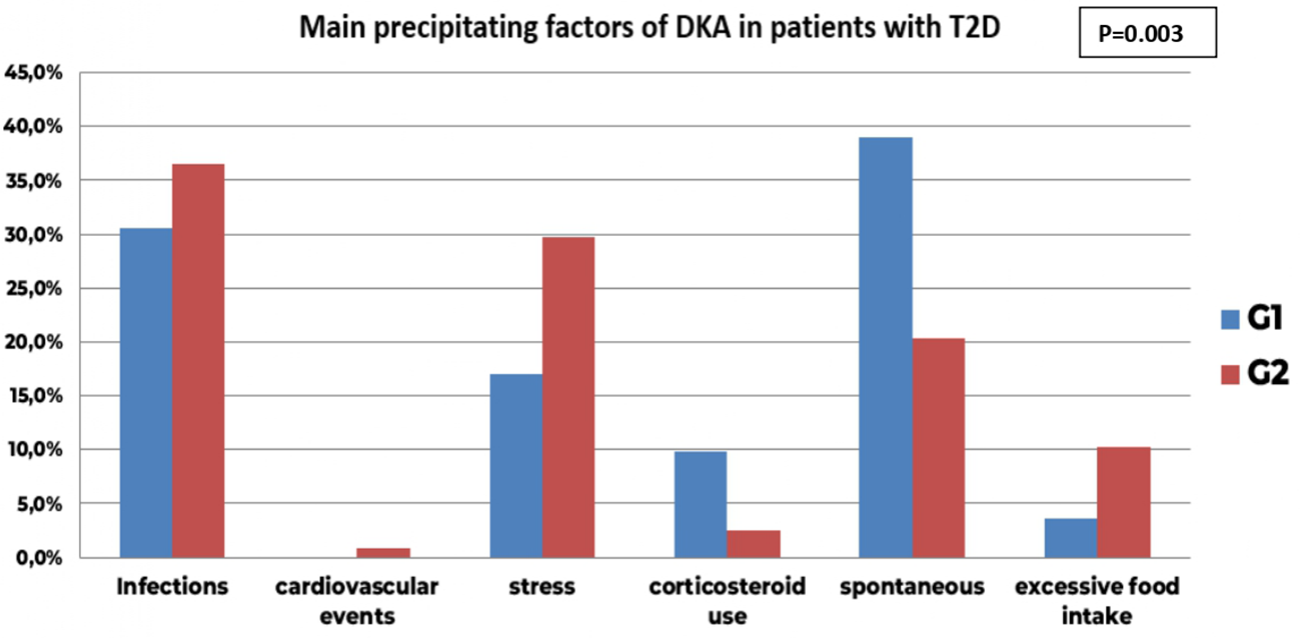
Comparison of main precipitating factors of DKA before (GI) and during COVID-19 pandemic in patients with new onset T2D (G2); the distribution of DKA precipitating factors in T2D differed significantly between the two groups (p=0.003) with a notable increase of stress (29.7% in G2 vs 17.1% in GI, AR-2) as well as excessive food intake notably hypertonic drinks (10.2% in G2 vs 3.7% in G1) at the expense of corticosteroid use as its accountability in precipitating DKA significantly decreased in G2 (2.5% in G2 vs 9.8% in GI, AR-2.2). The infectious precipitating factors remained comparable between the two groups (30.5% in G1 vs 36.4%, AR-0.9).

However, using subgroup analysis, the distribution of DKA precipitating factors in T2D differed significantly between the two groups (**p=0.003**) with a notable increase of stress (29.7% in G2 vs 17.1% in G1, AR=2) as well as excessive food intake notably hypertonic drinks (10.2% in G2 vs 3.7% in G1) at the expense of corticosteroid use as its accountability in precipitating DKA significantly decreased in G2 (2.5% in G2 vs 9.8% in G1, AR=-2.2) (**Figure 5**).

Even though the overall accountability of infectious causes did not differ between the two groups (36.4% in G2 vs 30.5% in G1, AR=0.9), infection sites had a significantly different distribution between the two groups (p<10^-3^) with COVID-19 becoming the first infectious precipitating factor of DKA in G2 (AR=4.2).

Time for DKA resolution was considerably higher in G2 14 ± 6.2 hours in G1 vs 16.5 ± 1.4 hours in G2, p=0.022

We found no significant difference in cumulative insulin dose required for DKA resolution in G1 78 ± 6.5 UI vs 82.5 ± 77 UI in G2, p=0.142.

Insulin dose at discharge was also comparable between the two groups (0.40 [0.29-047] UI/Kg/day in G1 vs 0.41 [0.28-0.50] in G2, p=0.895)

## Discussion

Since 2020, the COVID-19 pandemic has changed the world as we know it. It has led to changes in epidemiological and clinical presentations of various comorbidities such as DM and has been incriminated in the increasing trend of DKA (17).

The study of the temporal trend of hospital cases of DKA between March 2018 and February 2022 showed a significant upward trend.

This finding has important implications (**Table 2**). First, the incidence of DKA admitted in our department has been increasing well before COVID-19 pandemic.

**Table 2:** Different studies comparing the incidence of new onset DKA before and during COVID-19 pandemic.

Authors	Country	Year	Patients	Increased incidence of DKA	Type of DM
**Mastromauro et al.** (18)	Italy	2022	172	Yes (55% vs 36%)	T1D
**Jafari et al.** (19)	USA	2022	175	Yes	T1D
**Salmi et al.** (9)	Finland	2021	315	Yes (6.25 → 20)	T1D
**Vorgučin et al.** (20)	Serbia	2022	231	Yes (36.12 → 42.42)	T1D
**Khan et al.** (21)	USA	2022	14630	Yes	T2D, T1D
**Our study**	Tunisia	2023	340	Yes	T1D, T2D

This has long been corroborated all over the world namely in a cohort study by Zhong et al. where they studied the trends in hospital admission for DKA in adults in England between 1998-2013 and found a significant rise of DKA among adults with T1D as well as T2D (22). This has been attributed to the increase of the overall incidence of T2D, increased prevalence of infection and ketosis-prone T2D in minority groups (22).

The second key finding is that the incidence of DKA showed a sustained increase during the COVID-19 pandemic, showing a notable rise of almost 50% compared to a similar period prior to the pandemic. Our study also identified a statistically significant difference in the mean monthly incidence of DKA between the two periods, implying that the pandemic may have been the underlying factor contributing to this upward trend.

This observation is consistent with the findings of other studies (9, 18–21), which have reported a rising trend of DKA cases during COVID-19 pandemic. Indeed, an international multicenter study based on data from 13 national diabetes registries by Birkebaek et al. found a significant increase in the proportion of presentations for DKA, with a rise of 39.4% in 2020 and 38.9% in 2021. This increase exceeded the predicted year-on-year rise in prevalence, which was predicted to be 32.5% for 2020 and 33.0% for 2021 (6).

Many reasons could be behind this increase. The most apparent could be the delayed diagnosis of new onset DM due to the reluctance of individuals to seek medical attention for symptoms such as polyuria and polydipsia due to the fear of contracting the virus, leading to missed opportunities for earlier diagnosis. However, we found no significant difference in duration of polyuria and polydipsia preceding DKA in both study groups. This finding challenges the notion that delayed diagnosis has a significant influence on the increased incidence of DKA during the COVID-19 pandemic, arguably implying instead a direct effect of SARS-CoV-2 in the increased incidence of DKA during the pandemic.

Another possible reason is that SARS-CoV-2 may have caused direct damage to pancreatic beta cells, which could explain the observed increase in insulin-dependent diabetes and consequently in DKA cases, as hypothesized by Misra et al. (23).

The incidence of new onset DKA during COVID-19 pandemic according to pandemic waves shows a nadir during the third wave with a stronger re-increase during the fifth wave which saw the highest number of DKA case. The average monthly admission of DKA according to pandemic waves shows also a nadir during the third wave and a reincrease during the fifth wave without it being statistically significant (p=0.09).

Possible factors for the relative decline of DKA admitted in our department during the third wave include the relatively short duration of this wave, as defined by ONMNE, as well as strict social restrictions that were implemented during this wave. Moreover, during this particular wave, there was a simultaneous establishment of additional departments designated for the admission of COVID-19 patients, regardless of their DKA status. This occurrence potentially alleviated the burden on our department for a temporary period.

The pandemic variants of SARS-CoV-2 have been reported to change its viral characteristics. Mutations that alter the affinity of the virus to receptors may affect its ability to enter beta cells and cause cellular damage. For example, the Omicron variant is characterized by a higher ACE2 binding affinity (24). Therefore, it is plausible that the tropism for the pancreas differs according to SARS-CoV-2 variants (25). Although merely speculative, this can arguably explain the higher incidence of DKA during the fifth wave where Omicron variant was prevalent in Tunisia (26). However, data is insufficient to investigate whether the diabetogenicity of COVID-19 depends on its variants (25).

We found an increase of T1D incidence during COVID-19 pandemic which is in line with numerous studies around the world reported mainly among the pediatric population (9, 18–21, 27).

Although it was once believed that genetics played a significant role in T1D with over 50 genes identified, low concordance of T1D (<50%) in monozygotic twins implies that environmental factors, more specifically viruses, may be even more consequential than previously thought (28). Respiratory viruses have also been incriminated as shown by a large Norwegian cohort study published in June 2018 by Ruiz et al. who have investigated the risk of new onset DM subsequent to Influenza A (H1N1) pandemic in June 2009 and reported a twofold increased risk of new onset T1D (16).

The molecular mimicry hypothesis is the most appealing pathophysiological pattern mediating beta cells autoimmune injury as suggested by Andrade et al. in a study published in October 2022 where amino-acid sequences of human insulin and GAD65 as long as their epitopes were compared with the sequences of the SARS-CoV-2 proteins (S protein, Spike protein) (29). Epitope similarity between human insulin and SARS-CoV-2 and between GAD65 and SARS-CoV-2 ranged between 45 to 60%. This would plausibly result in the development of an immune cross-reaction to self-antigens, thus triggering T1D (29). However, without clinical data from individuals with T1D or COVID-19, it is difficult to establish a direct causal relationship between SARS-CoV-2 and the triggering of T1D.

The other related mechanism is virus-induced beta cell injury causing the release of sequestered antigens which would eventually be expressed by antigen-presenting cells increasing the risk of autoantibodies generation (30).

Although we found no significant difference between T1D frequencies before and during COVID-19 pandemic, autoantibodies such as Anti-GAD and Anti-IA2 titers interestingly increased during the pandemic period compared with the pre-pandemic period (p=**0.021**), (p=**0.009**) respectively. This would reasonably incriminate SARS-CoV-2 in triggering an auto-immune insulitis. Similarly, Wang et al. reported a marked increase in autoantibody reactivities in COVID-19 patients as compared to uninfected individuals reflected by a higher prevalence of autoantibodies against immunomodulatory proteins (including cytokines, chemokines, complement components and cell-surface proteins) (31). Even though this study also discusses the presence of tissue-associated autoantibodies in patients with COVID-19, targeting various organs and systems including vascular cells, coagulation factors, platelets, connective tissue, extracellular matrix components, anti-islet antibodies have not been investigated. Therefore, we cannot establish with certainty a direct link between COVID-19 and T1D.

We found an increase of new onset T2D cases by 44% during COVID-19 pandemic.

Insulin resistance is suspected to be directly induced by SARS-CoV-2 as speculated by a study by Montefusco et al. where it was found that compared to healthy controls, patients with COVID-19 had significantly higher levels of mean fasting insulin, proinsulin, and C-peptide, as well as higher values of the homeostasis model assessment of beta cell dysfunction (HOMA-B) and homeostasis model assessment of insulin resistance (HOMA-IR) which were correlated with inflammation markers indicating that COVID-19-related insulin resistance has an inflammatory basis suggesting that insulin resistance and beta cell dysfunction in COVID-19 may be triggered by a proinflammatory environment initiated by a cytokine storm (12).

The increase of accountability of stress in precipitating DKA can be explained by the levels of psychological distress associated with COVID-19 pandemic. Indeed, Xiong J et al. conducted a systematic review showing high levels of anxiety-related symptoms, depression, posttraumatic stress disorder, psychological distress in the general population in various countries during the COVID-19 pandemic. This can be explained by periods of lockdown where unemployment and marital problems peaked, not to mention frequently being exposed to worrisome news about COVID-19 (32).

The increased responsibility of excessive food intake as a trigger for DKA may be ascribed to modifications in eating behavior. According to a systematic review of longitudinal studies which compared eating habits before and during COVID-19 pandemic, an increased tendency towards eating snacks and a preference for sweets and ultra-processed food rather than fruits and vegetables was reported (33).

Among infectious precipitating factors, COVID-19 became the first infectious precipitating factor of DKA in G2 with a decrease of the accountability of pulmonary and other influenza like illness, the latter is likely due to the enforcement of COVID-19 public health protocols leading to a decline in the spread of common respiratory viruses (34).

DKA was managed classically by hydration, insulin infusion along with electrolyte supplementation. The time it took for DKA to resolve was considerably higher during COVID-19 pandemic compared to the prepandemic period (p=0.022). This supports the findings of Farzadfar et al. where longer time to DKA resolution was found in patients infected with COVID-19 (35).

This can be explained by various strategies implemented during the COVID-19 pandemic to effectively reduce the risk of healthcare workers being exposed to the virus while providing care to patients with COVID-19 (35). These strategies involved minimizing the use of venous insulin infusions for DKA whenever possible and decreasing the frequency of capillary glycemia checks for patients on subcutaneous insulin regimens (35). These measures underscored the urgent need for innovative technologies such as continuous glucose monitoring (CGM), especially in the context of a pandemic, as they would enable healthcare exposure to be minimized.

Regrettably, implementing such strategies is challenging in resource-limited settings, particularly in financially constrained regions like certain areas in Africa.

Our study is subject to several limitations that should be acknowledged. Firstly, it is a retrospective study, conducted at a single center, which may restrict the generalizability of our findings. Additionally, the absence of computerized national registries for new onset DKA in our country limits our ability to access comprehensive data and needs manual collection from medical records.

Another important limitation is the challenge of accurately establishing the history of COVID-19 infection in our study population. While we documented cases of SARS-CoV-2 infection at the time of diagnosis, differentiating between a prior infection and a vaccinal status was not possible particularly as our study was conducted during a period when COVID-19 vaccinations became widely accessible.

Despite these limitations, our study provides valuable insights into the incidence and potential factors associated with DKA in our specific context of an African country. We believe that our findings contribute to the existing knowledge base and can serve as a foundation for future research efforts aimed at addressing these limitations and expanding our understanding of the relationship between COVID-19 and DKA.

## Conclusion

The COVID-19 pandemic has had significant impacts on the epidemiology of DKA. While the increasing trend in DKA cases was observed even prior to the pandemic, the COVID-19 period has seen a significant rise in DKA incidence, which may be due to delayed diagnosis or arguably *via* an auto-immune triggering mechanism or an inflammatory milieu favorable for insulin resistance.

The potential long-term implications of these trends are concerning, particularly in terms of the burden on healthcare systems and the increased risk of complications associated with these conditions. As such, it is important for healthcare providers, particularly in Africa, to remain vigilant in monitoring and treating patients with DM, especially during times of crisis such as the COVID-19 pandemic.

## Data availability statement

The raw data supporting the conclusions of this article will be made available by the authors, without undue reservation.

## Ethics statement

Written and informed consent was obtained from the patients for publication of the submitted article. The anonymity and data confidentiality of the patients’ data were respected. Approval from the Ethical Committee of University of Medicine of Sousse was obtained under the assigned number 4935/2023.

## Author contributions

AG and TA drafted the manuscript. All authors have contributed significantly to this work, providing substantial intellectual input, and have thoroughly reviewed and approved the final manuscript.
